# Zn Complex with Homovanillic Acid: Theoretical (B3LYP/6-311++G(d,p)), Structural (FT-IR, NMR), Thermal (TG, DTG, and DSC) and Biological (Antioxidant and Antimicrobial) Characteristics

**DOI:** 10.3390/ma18102374

**Published:** 2025-05-20

**Authors:** Mariola Samsonowicz, Monika Kalinowska, Adriana Dowbysz, Kamila Koronkiewicz, Bożena Kukfisz, Anna Pietryczuk

**Affiliations:** 1 Department of Chemistry Biology and Biotechnology, Bialystok University of Technology, Wiejska 45E, 15-351 Bialystok, Poland; m.kalinowska@pb.edu.pl (M.K.); adriana.dowbysz@pb.edu.pl (A.D.); kamila.koronkiewicz@pb.edu.pl (K.K.); 2 Institute of Safety Engineering, Fire University, Slowackiego Street 52/54, 01-629 Warsaw, Poland; bkukfisz@apoz.edu.pl; 3 Department of Water Ecology, Faculty of Biology, University of Bialystok, Ciołkowskiego 1J, 15-245 Bialystok, Poland; annapiet@uwb.edu.pl

**Keywords:** homovanillic acid, metal complex, zinc, antioxidant assay, spectroscopic characterization

## Abstract

In this study, the structure of the synthesized Zn(II) complex with homovanillic acid (HVA) was investigated using the FT-IR, UV/Vis, and NMR spectroscopic methods, as well as elemental and thermal (TG, DTG, and DSC) analysis. The stoichiometric molar ratio of metal:ligand for the solid form of the complex was established as 1:2, with coordination through the carboxylate group and aromatic ring substituents. The theoretical structural and electronic parameters were calculated by the use of the B3LYP/6-311++G(d,p) method. Antioxidant properties were examined using spectroscopic tests: DPPH (1,1-diphenyl-2-picrylhydrazyl radical), FRAP (ferric reducing antioxidant activity), and ABTS (2,2′-azino-bis-(3-ethylbenzothiazoline-6-sulfonic acid) (diammonium salt radical cation). The Zn(II) complex with HVA showed similar or lower antioxidant properties compared to the ligand, depending on the antioxidant assay. The antimicrobial activity of acid and its complex with Zn(II) against *Escherichia coli*, *Bacillus subtilis*, and *Candida albicans* were also investigated by evaluation of the minimum inhibitory concentration (MIC). The Zn(II) complex shows higher antibacterial and antifungal activity compared to HVA.

## 1. Introduction

**Figure 1:**
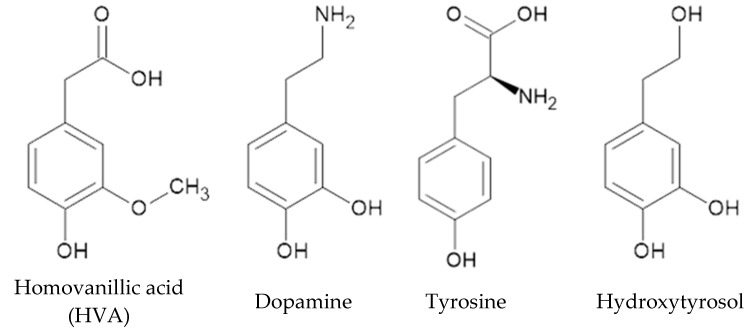
Structural formulas of homovanillic acid and its precursors.

Homovanillic acid (HVA) or 2-(4-hydroxy-3-methoxyphhenyl)acetic acid (Figure 1) is one of the metabolites of dopamine, a neurotransmitter produced from tyrosine in the peripheral endocrine system and central nervous system [1,2]. Dopamine is responsible for the control of posture, movement, attentiveness, and mood in the face of various stresses affecting humans [2]; it has a similar function in plants [3]. The tyrosine derivative, hydroxytyrosol, present in olives, is one of the sources of homovanillic acid in the human body [4]. Its metabolism occurs in the intestines, by glucuronidation, sulfation, and methylation-oxidation involving enzymes. HVA’s role as a neurotransmitter and dopamine marker has been described [5,6]. As a marker of metabolic stress, it is used to detect oxidative enzymes (e.g., peroxidase, glucose oxidase, and xanthine oxidase) [7].

One of the natural sources of homovanillic acid is olives. Depending on the variety and form of occurrence, Bianco and Uccella determined the HVA content, which ranged from 3 to 4 mg soluble HVA/100 g of fresh olive pulp, 13–74 mg soluble-esterified HVA/100 g, and 22–37 mg of insoluble-bound HVA/100 g [8], and, consequently, HVA is also present in olive oil [1]. HVA was also found to be present in wort (0.482 µg/mL) and Italian lager beer (0.58 µg/mL) [9], and in raw and fermented hemp seeds, respectively 9.2 and 57.9 µg/mL [10]. HVA supplementation by the oral route can also be supported by diet reach in nuts [11] or cocoa extract [12]. An additional way to increase the absorbed intake of this valuable component is through probiotic supplementation, such as in a form of fermented teas. Mihai et al. tested several types of fermented teas (known as kombucha), of which they found a high level of HVA in Ecuadorian horchata tea, at 74.45 mg/100 g dry weight [13].

Homovanillic acid shows significant anti-inflammatory properties. In their work, Shan et al. determined the anti-inflammatory activity of fermented hemp seeds, for which the homovanillic acid and indolelactic acid were mainly responsible [10]. The anti-inflammatory activity of extracts was examined against inflammatory cytokines: TNF-α, IL-6, IL-1β, and NO. In addition, the authors examined their activity in a simulated digestive system and HVA bioaccessibility. They observed an increase in HVA activity after entering the intestines and stomach, and closely linked the relationship of counteracting inflammatory processes to the amount of HVA. They concluded that oral consumption of hemp seeds (especially fermented ones) allows absorption of about 60% of anti-inflammatory substances. The authors proposed this plant material as an ingredient in a potential product targeting the prevention of conditions related to inflammatory processes in the digestive system. Ibero and co-researchers, on the other hand, reported an alleviation of depressive symptoms with an increased supply of HVA in the diet of obese individuals [12]. This was because the level of HVA in the blood also determined its presence in the peripheral nervous system. This is how HVA differs from dopamine—being able to cross the blood–brain barrier.

There are reports of good antioxidant activity of homovanillic acid and its derivative, homovanillyl alcohol. However, their precursor, the aforementioned hydroxytyrosol, as a simple ortho-phenol showed higher activity [14]. Tuck and Hayball examined the activity of HVA, homovanillyl alcohol, and hydroxytyrosol against the DPPH^•^ radical, where the IC_50_ values were 14.8, 11.4, and 2.4 μM, respectively [4].

Metal cations play a very important role in various biological systems. They can modify the biological properties of important natural ligands and affect the therapeutic properties of some compounds that are used as drugs [15]. Some metal complexes show higher antioxidant activity compared to ligands alone, which may be due to stabilization of semiquinone radicals by some metal cations or different kinetics of the reaction of ligands and their complexes with radicals. This may also be due to the participation of the metal center in the reaction with radicals as well as changes in the redox potential and thermochemical parameters during these reactions [16]. There are also examples from the literature that complexes of some ligands with metals have lower antioxidant properties than the ligands themselves.

The motivation to undertake the research presented in this paper was to search for more effective antioxidants that can be applied as active agents in pharmacy and medicine. The presented research is part of a larger project in which we are analyzing the effect of metal ion complexation on the biological properties of ligands with natural origin [17,18,19,20,21,22]. Furthermore, plant phenolic compounds and their metal complexes are currently being intensively investigated as biologically active compounds with effective antimicrobial (antibacterial and antifungal) and antioxidant properties. The changes in the structural parameters of ligands after metal ion complexation affect the biological properties of the formed complex, including antioxidant and antimicrobial activity. Knowledge of the structure of a chemical compound based on the experimental (e.g., FT-IR, UV/Vis, ad NMR) data and quantum mechanical calculations (optimization of molecular structure, electronic charge distribution, HOMO-LUMO gap, etc.) allows for the understanding of the relationship between the molecular structure and biological activity of the compound.

In this work, the Zn(II) complex with 2-(4-hydroxy-3-methoxyphenyl)acetic acid (Zn-HVA) was synthesized in a solid state and studied by means of elemental, thermogravimetric, NMR, and FT-IR analyses. The composition of the metal complex in aqueous solution was established by the spectrophotometric UV/Vis method. The antioxidant activity of homovanillic acid (HVA) and its complex with Zn(II) was also studied by means of a ferric reducing power assay (FRAP) as well as in the reactions with DPPH^•^ and ABTS^•+^ cation radicals. The molecular structures of homovanillic acid and the zinc(II) complex were studied by means of quantum chemical calculations at the B3LYP/6-311++G(d,p) level and are discussed in the context of the antioxidant activity of these compounds. Moreover, the antimicrobial properties of homovanillic acid and the Zn(II) complex toward *Escherichia coli*, *Bacillus subtilis*, and *Candida albicans* were determined, and the effect of metal ion on the antimicrobial potential of the ligand is discussed.

## 2. Materials and Methods

### 2.1. Materials

Homovanillic 2-(4-hydroxy-3-methoxyphenylacetic) acid, sodium hydroxide, zinc chloride (ZnCl_2_·2H_2_O), acetic acid, sodium acetate, 2,4,6-tris(2-pyridyl)-s-triazine (TPTZ), iron chloride (FeCl_3_·6H_2_O), potassium persulfate, 2,2-diphenyl-1-picrylhydrazyl (DPPH), diammonium 2,2′-azino-bis(3-ethylbenzothiazoline-6-sulfonate) (ABTS), potassium peroxodisulfate, phosphate buffer, methanol, ethanol, and hydrochloric acid were purchased from Sigma-Aldrich Co. (St. Louis, MO, USA) and used without purification.

### 2.2. Synthesis

A mass of 0.3 g (weighed to 5 decimal places) of homovanillic acid was added to an appropriate volume of 0.100 M NaOH aqueous solution in a molar stoichiometric ratio of 1:1. The solution was slowly concentrated at 70 °C to approximately 30% of the initial volume. Then, an appropriate volume of 0.500 M ZnCl_2_ aqueous solution was added to the solution so that the ligand was in excess. The occurred precipitate was filtered and washed several times with distilled water until no chloride ions were found in the filtrate based on the reaction with AgNO_3_ (0.1 M). Then, the precipitate was dried in a laboratory dryer at 105 °C for 24 h.

### 2.3. NMR Spectra

^1^H NMR and ^13^C NMR spectra of the DMSO sample solution of the HVA and Zn complex were recorded with a Bruker Avance II 400 MHz unit (Bremen, Germany) at a room temperature with TMS as an internal reference.

### 2.4. FT-IR Spectra

The FT-IR spectra of HVA and the Zn-HVA complex in the solid state were recorded with a Cary 630 FTIR Agilent Technologies spectrometer (Santa Clara, CA, USA), using the KBr pellet matrix technique, within the range of 400–4000 cm^−1^. The resolution was 1 cm^−1^.

### 2.5. UV-Vis Study

The metal ion: ligand molar ratio in an aqueous solution for the Zn-HVA complex was determined by the spectrophotometric mole-ratio method. The spectra in the range of 200–550 nm were recorded for solutions with a constant mole number for HVA and a varied volume of zinc(II) ions using an Agilent Cary 5000 spectrophotometer (Agilent, Santa Clara, CA, USA). The concentration of HVA was 0.5 mM, while the concentration of ZnCl_2_ changed from 0 to 0.20 mM. All solutions were prepared in Tris-HCl buffer (pH = 7.4; C = 50 mM). The experiment was performed in three independent experiments.

### 2.6. Elemental and Thermogravimetric Analyses

Elemental analysis for the mass percentages of carbon and hydrogen was performed with the Perkin Elmer 2400 equipment (Waltham, MA, USA). The thermal behavior of the Zn-HVA complex was investigated using a 209 F1 Libra^®^ Netzsch thermogravimeter (Selb, Germany). Tested samples of 10 ± 0.1 mg were placed in ceramic crucibles and heated from 25 to 800 °C at a heating rate of 10 °C/min under the air atmosphere.

### 2.7. Antioxidant Activity

#### 2.7.1. Ferric Reducing Antioxidant Power Assay (FRAP)

A FRAP working solution was prepared by mixing 300 mM of acetate buffer, 10 mM of TPTZ solution in 40 mM of HCl, and 20 mM of FeCl_3_ in the ratio 10:1:1 (*v*/*v*). The calibration curve was prepared for the series of FeSO_4_ solutions within the concentration range of 0.300–0.050 mM. Then, 3.0 mL of the FRAP solution was added to the test tubes with 0.4 mL of the selected concentration of the standard solution of FeSO_4_. The ferric reducing activity of the studied compounds was determined by adding to the test tubes 3.0 mL of the FRAP solution and 0.4 mL of the Tris-HCl solution of the tested compounds with the concentration: 25 or 50 µM. The Tris-HCl solution of HVA was prepared by adding the Tris-HCl solution of HVA (C = 0.1 mM; 5.0 or 2.5 mL) into a volumetric flask with a volume of 10 mL and refilling to the mark with Tris-HCl solution. The Tris-HCl solution of Zn-HVA was prepared in a similar way, i.e., the Tris-HCl solution of HVA (C = 0.1 mM; 5.0 or 2.5 mL) and ZnCl_2_ (C = 1 mM; 0.50 or 0.25 mL) were added into a volumetric flask with a volume of 10 mL and refilling to the mark with Tris-HCl solution. After 8 min of incubation, the absorbance of each sample was measured at the 595 nm wavelength. Reducing activity was expressed as Fe^2+^ equivalents [µM] on the basis of the obtained calibration curve for FeSO_4_ (y = 33.90x − 0.002; R^2^ = 0.999). The assay was performed in five repetitions in three independent experiments, according to the procedure described in [20].

#### 2.7.2. DPPH^•^ Antiradical Activity Assay

The methanolic solution of DPPH^•^ (60 µM) and Tris-HCl solutions of HVA (0.1 mM) and zinc complex of HVA (0.1 mM) were prepared before the experiment in a similar way as described above. The appropriate volumes of the solutions of HVA or Zn complex of HVA were added to 2 mL of DPPH^•^ and adjusted with Tris-HCl to the volume of 3 mL. The control probe consisted of Tris-HCl and DPPH^•^. The samples were incubated for 1 h, and then the absorbance was measured at λ = 516 nm, using an Agilent Carry 5000 (Santa Clara, CA, USA) spectrophotometer. The percentage of inhibition of DPPH^•^ was calculated according to the following formula:

$$\%I=\frac{A_{control}^{516}-A_{sample}^{516}}{A_{control}^{516}}\cdot 100\%\;\left[\%\right]$$

where $A_{control}^{516}$ is the absorbance of the control sample, and $A_{sample}^{516}$ is the absorbance of the tested sample. The assay was performed in five repetitions in three independent experiments, according to [23].

#### 2.7.3. ABTS^•+^ Antiradical Activity Assay

The ABTS water solution (5.4 mM) and K_2_S_2_O_8_ water solution (1.74 mM) were prepared and mixed in a volume ratio of 1:1 (*v*/*v*). After 12 h incubation with no light, the radical cation ABTS^•+^ was produced. The mixture was diluted with methanol to obtain a solution with an absorbance of ca. 0.8 at λ = 734 nm. Then, 1.5 mL of Tris-HCl solutions of HVA or Zn-HVA at the concentration range of 0.0013–0.005 mM and 1.5 mL of diluted ABTS^•+^ solution (0.01 mM) were incubated in glass test tubes. The control probe consisted of 1.5 mL of Tris-HCl and 1.5 mL of diluted ABTS^•+^ solution (0.01 mM). The samples were incubated for 7 min, and then the absorbance was measured at λ = 734 nm, using an Agilent Carry 5000 (Santa Clara, CA, USA) spectrophotometer. The percentage of inhibition of ABTS^•+^ was calculated according to the following formula:

$$\%I=\frac{A_{control}^{734}-A_{sample}^{734}}{A_{control}^{734}}\cdot 100\%\;\left[\%\right]$$

where $A_{control}^{734}$ is the absorbance of the control sample, and $A_{sample}^{734}$ is the absorbance of the tested sample. The assay was performed in five repetitions in three independent experiments, according to [24].

### 2.8. Antimicrobial Activity

The MIC was determined by serial dilutions of the test compound in an agar medium to which an appropriate microbial inoculum was then added and incubated. The microorganisms used in the studies were from the Polish Collection of Microorganisms (Wrocław, Poland): *E. coli* (PCM 2857), *B. subtilis* (PCM 2850), and *C. albicans* (PCM 2566-FY). The bacteria were grown overnight and then resuspended in physiological saline to an optical density of 600 nm (OD = 600) of 0.60, corresponding to 5.0 × 10^8^ CFU/mL. The bacteria (0.1 mL of reconstituted suspension) were plated on sterile Mueller-Hinton agar plates (Oxoid, CM0337B), to which appropriate amounts of the test compounds had been previously added to obtain the desired concentration. The tested solid samples of the studied compounds were dissolved in DMSO solution (Sigma-Aldrich, 276855) and distilled water so that the concentration of DMSO did not exceed 5%. The following concentrations of compounds were taken into consideration: 1000, 950, 900, 850, 800, 750, 700, 650, 600, 550, 500, 450, 400, 350, 300, 250, 200, 150, 100, and 50 ug/mL. The negative control was agar plates to which 5% DMSO had been added, and the positive control was plates with gentamicin (Sigma-Aldrich, 345815) (for bacteria) or flucanazole (Sigma-Aldrich, F-031) (for fungi). The plates were incubated at 37 °C for 24 h. The lowest concentration without visible bacterial growth was defined as the MIC (minimal inhibitory concentration). The experiment was performed in triplicate.

### 2.9. Computational Details

All calculations were performed using the Gaussian09 Revision D.01 software package [25]. Visual representation of the results of the theoretical calculations was realized using GaussView 6 software (Gaussian Inc., Wallingford, CT, USA). The B3LYP/6-311++G(d,p) hybrid Density Functional Theory (DFT) method was used to determine the optimized geometric structures of the HVA and Zn-HVA and corresponding radicals, anions, and radical cations.

In the case of theoretical description of phenolic structures, mainly methods based on the Density Functional Theory (DFT) are used. Many works provide evidence of good agreement between experimental and theoretical results obtained using the DFT method. On the other hand, the use of a hybrid functional method (e.g., B3LYP) in combination with the 6-311++G (d,p) basis set allowed, in many studies, accurate description of the reactivity of various groups of polyphenols. In our study, we used the B3LYP/6-311++G (d,p) method to study the structure of the HVA and Zn(II) complex, because the results of many of our works have proven high agreement between the experimental and theoretical data. Moreover, the use of the same computational method in many of our previous works allows us to analyze trends in changes in the structure of biologically active ligands under the influence of the metal complexation [5,17,18,21,26].

Electron population analysis was carried out using the natural bonding orbitals (NBOs) [27]. The condensed Fukui indices [28] and Fukui function dual descriptor [29] were calculated using the natural charges (NBOs) on the basis of the equations described in the literature. The selected reactivity descriptors [21], such as the ionization potential, electron affinity, electronegativity, chemical hardness and softness, and electrophilicity index were calculated on the basis of the values of the energy of HOMO (highest occupied molecular orbital) and LUMO (lowest unoccupied molecular orbital). In addition, BDE (bond dissociation enthalpy), IP (ionization potential), PA (proton affinity), PDE (proton dissociation enthalpy), and ETE (electron transfer enthalpy) of homovanillic acid and its complex with Zn were calculated in the gas phase for 298.15 K and 1.0 atmospheric pressure. The values of the mentioned parameters were determined as follows [2]:

Hydrogen atom transfer (HAT) mechanism:

$$R^{•}+\mathrm{ArOH}\;\to \mathrm{RH}+{\;\mathrm{ArO}}^{•}$$

$$\mathrm{BDE}=H({\mathrm{ArO}}^{•})\;+\;H(H^{•})\;-\;H(\mathrm{ArOH})$$

Single electron transfer followed by proton transfer (SET-PT) mechanism:

$$R^{•}+\mathrm{ArOH}\to R^{-}+{\mathrm{ArOH}}^{•+};\;{\mathrm{ArOH}}^{•+}+R^{-}\to {\mathrm{ArO}}^{•}+H^{+}$$

$$\mathrm{IP}=H({\mathrm{ArOH}}^{•+})\;+\;H(e^{-})\;-\;H(\mathrm{ArOH})$$

$$\mathrm{PDE}=H({\mathrm{ArO}}^{•})\;+\;H(H^{+})\;-\;H({\mathrm{ArOH}}^{•+})$$

Sequential proton-loss electron transfer (SPLET) mechanism:

$$\mathrm{ArOH}\to {\mathrm{ArO}}^{-}+H^{+}$$

$${\mathrm{ArO}}^{-}+R^{•}\to {\mathrm{ArO}}^{•}+R^{-}$$

$$R^{-}+H^{+}\to \mathrm{RH}$$

$$\mathrm{PA}=H({\mathrm{ArO}}^{-})\;+\;H(H^{+})\;-\;H(\mathrm{ArOH})$$

$$\mathrm{ETE}=H({\mathrm{ArO}}^{•})\;+\;H(e^{-})\;-\;H({\mathrm{ArO}}^{-})$$

The calculated gas-phase enthalpy for the proton (H(H^+^)), electron (H(e^−^))and hydrogen atoms (H(H^•^)) were taken from the literature and were equal to 6.197 kJ/mol, 3.146 kJ/mol [30,31], and −1306 kJ/mol, respectively.

The results were used to compare the antioxidant capacity of HVA and Zn-HVA, as well as to assess whether the hydrogen atom transfer mechanism or electron donation mechanism has the greatest impact on the antioxidant activity of the analyzed compounds.

## 3. Results

### 3.1. Quantum Chemical Calculations

**Figure 2:**
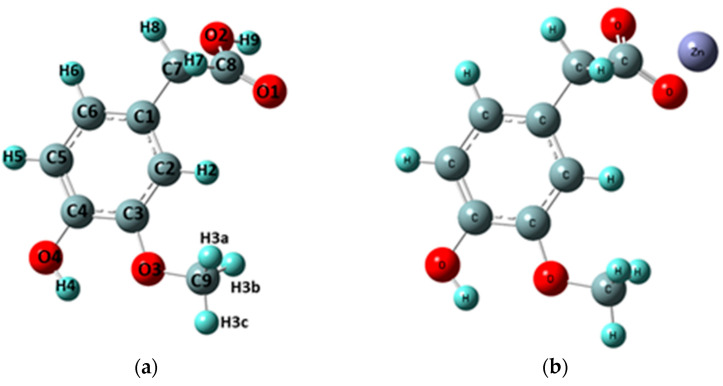
Optimized structures of: (**a**) HVA acid [5] (with atom numbering); (**b**) Zn complex molecules.

The optimized structure at B3LYP/6-311++G(d,p) of homovanillic acid and its complex of zinc are presented in Figure 2. The initial structure for the calculations was the structure of homovanillic acid presented by Samsonowicz et al. [5].

Using specifically the mole-ratio method, it was found that in the aqueous solution at pH = 7.4, homovanillic acid forms a complex with Zn(II) in a 1:1 molar ratio. The same molar ratio of ligand:metal 1:1 was used to prepare the solution of the studied compounds for the antioxidant assays. Therefore, the calculations were performed for the complex with stoichiometry of ligand:metal 1:1 (Figure 2b) as well. The calculated complex (Zn-HVA) was a cation (+1) in which the presence of a counterion (Cl^−^) and water molecules were not taken into account.

#### 3.1.1. Changes in Bond Lengths and Angles

The calculated optimized geometric parameters (bond lengths and bond angles) for homovanillic acid and Zn(II) complex are summarized in Tables S1 and S2 (in the Supplementary Materials), respectively. Table 1 presents the bonds between the atoms whose lengths changed (after the formation of the Zn-HVA complex) by more than 0.005 Å and the angles between bonds whose values changed by more than 1.

The coordination of zinc by the carboxylate group mainly affected the geometry of the C-CH_2_-COOH^−^ moiety. After the formation of the Zn-HVA complex, no major changes were observed in the bond lengths and angles between the carbon atoms in the aromatic ring. The most pronounced changes were observed in the geometry of the carboxylic/carboxylate group. Coordination of Zn(II) through the carboxylate group caused the extension of the C8-O1 bond and the shortening of the C8-O2 bond; as a result, the lengths of these bonds became almost equal. The bond length between the O2-H9/Zn atoms in the formed Zn complex increased in comparison to the ligand molecule from 0.970 Å to 1.150 Å, while the O1-h9/Zn bond length decreased from 2.291 Å to 2.128 Å. The greatest changes in bond lengths in relation to the acid were observed for the C7-C8-O1-O2 system (Table 1 and Table S1).

The formation of the Zn(II) complex also caused changes in the size of the angles between the atoms in the carboxylate group. The size of the C8-O1-9H/Zn angle significantly increased by about 33°, and the size of the C8-O2-9H/Zn angle significantly decreased by 19°. In this case, equalization of the sizes of both angles was also observed, and they were 88.32° and 88.73°, respectively. The C7-C8-O2 angle increased and the C7-C8-O1 angle decreased in the complex compared to the homovanillic acid molecule, by 7.78° and 5.72°, respectively. Compared to the ligand, small changes in angles of about 0.1–0.7° were observed: an increase in the size of the angles C7-C1-C2, C1-C7-C8,C7-C1-H8 and H7-C7-C8, with a decrease in the angles C6-C1-C7 and C1-C7-7H.

#### 3.1.2. Natural Bond Orbital (NBO)

The NBO (Natural Bond Orbital) atomic charges calculated for the studied theoretically optimized molecules are shown in Table 2 (selected) and Table S3. Under the influence of the Zn-HVA complex formation, the distribution of the electron charge in the HVA molecule changes. The changes mainly concern the carboxylate group, on which the negative charge distinctly increases (by 0.235e), as do the carbon atoms: C7 and C8 (from CH_2_ group). However, the formation of the complex did not affect the charge distribution in the aromatic ring and the hydroxyl and methoxy groups associated with the aromatic ring. The electron density around the O1 and O2 atom increased, while the electron density on the C7 and C8 carbon atoms decreased compared to the acid.

#### 3.1.3. HOMO-LUMO Analysis

**Figure 3:**
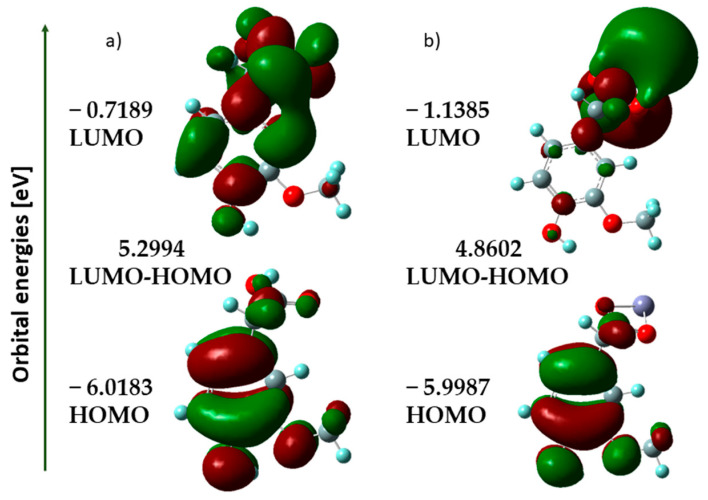
HOMO and LUMO energy orbitals of (**a**) HVA; (**b**) Zn-HVA.

It is well known that the energies of HOMO and LUMO orbitals are very useful for qualitative descriptions of biological activity and reactivity of a molecule [18]. The highest occupied molecular orbital (HOMO) can be used to determine the electron-donating ability of a molecule. As many studies show, the antioxidant activity of phenolic compounds depends on the energy of HOMO orbitals. With an increase in HOMO value and decrease in ionic potential (IP) value, the molecule shows an increasing tendency to donate electrons [32]. The HOMO and LUMO maps of homovanillic acid and its cation complex (+1) with Zn are shown in Figure 3. For the theoretical structures of HVA and Zn-HVA, the shape, position, and energy of the HOMO and LUMO orbitals were determined. The calculated results show that the HOMO energy of Zn(II) complex is slightly higher (−5.9987 eV) than for acid (−6.0183 eV) indicating that Zn-HVA has a slightly stronger electron-donating ability than the ligand alone.

This would indicate that the formed complex should exhibit higher antioxidant properties than the ligand; however, experimental studies show that homovanillic acid has a slightly higher antioxidant potential. This is probably due to the fact that both HVA and Zn-HVA have very similar values for the HOMO orbital energy and ionization potential, and the difference between the energy of HOMO orbitals is 0.0196 eV.

Chemical reactivity and bioactivity of molecules can also be characterized by the ΔE value (the energy difference between the HOMO and LUMO orbitals). A lower HOMO-LUMO gap indicates less stability and higher reactivity, while a higher gap means the reverse case. In the case of Zn-HVA, this gap is lower compared to HVA, which may indicate greater reactivity of the complex.

Based on the energies of the HOMO and LUMO orbitals, general reactivity descriptors were calculated and are collected in Table 3. Parameters such as the ionization potential, electron affinity, electronegativity, electronic chemical potential, chemical hardness, chemical softness, and electrophilicity index were used to predict electronic charge transfer in the molecule and reactivity and stability of the compounds.

Electron affinity measures the energy change when an electron is added to the molecule. The electron affinity for Zn-HVA (1.139 eV) was higher than that of acid alone (0.719 eV), indicating that Zn-HVA has a higher tendency to accept an electron.

Electronegativity (χ), in turn, describes the atom’s ability to attract electrons. The electronegativity value for acid (3.369 eV) was lower than that of Zn-HVA (3.569 eV), indicating a decreased electron-attracting ability in the acid. The chemical hardness and chemical softness values provide information about the stability and reactivity of molecules. Zn-HVA exhibited lower hardness (2.430 eV) and higher softness (0.206 eV) in relation to acid alone, suggesting increased reactivity.

The electrophilicity index (ω) describes the tendency of a molecule to accept electrons. Zn-HVA showed a higher electrophilicity index than acid, indicating its higher electron accepting capacity.

#### 3.1.4. Thermodynamical Parameters

Descriptors related to the free radical scavenging abilities of investigated compounds were determined: bond dissociation enthalpy (BDE—the HAT mechanism), proton affinity and electron transfer enthalpy (PA and ETE—the SPLET mechanism), and ionization potential and proton dissociation enthalpy (IP and PDE—the SET-PT mechanism). The thermodynamic parameters for the analyzed compounds are summarized in Table 3.

Bond dissociation enthalpy (BDE) of the phenolic OH group is a measure of antioxidant power (free radical scavenging capacity) of phenolic compounds. The BDE energy value parameter describes the ability to donate H atoms. A lower value of BDE is associated with higher antioxidant potency.

Homovanillic acid had a lower BDE value (95.16 kcal/mol) than its complex with Zn(II) (104.31 kcal/mol), suggesting the higher antioxidant potential of HVA compared to Zn-HVA. This has been confirmed in experimental studies with the DPPH^•^ radical and ABTS^•+^. The obtained values (for both HVA and Zn-HVA) were higher than the OH BDE values, which correspond to effective antioxidants (i.e., below 77–85 kcal/mol [33]).

Ionization potential (IP) is related to electron loss and the formation of the respective radical cation. Molecules with lower IP are readily engaged in chemical reactions with radical species [2]. This describes the first step of the SET-PT mechanism. The data collected in Table 3 show that Zn-HVA can enter into reactions with radicals slightly more easily than HVA, although the IP difference between the acid and the complex is small (about 17 kcal/mol).

The PDE parameter, which describes the second step of the SET-PT mechanism, was much lower for HVA than the Zn-HVA, which suggests that HVA contributes to the second step of SET-PT to a greater extent than the complexes of Zn-HVA.

The PA and ETE values are the enthalpy of the reaction related to the SPLET mechanism. Both compounds have similar proton affinity (PA) values of 359.67 and 344.75 for HVA and Zn-HVA, respectively. Similarly, taking into account the ETE parameter, the values do not differ much and are 49.59 for the acid and 73.67.00 kcal/mol for the complex with Zn(II).

#### 3.1.5. Molecular Electrostatic Potential

**Figure 4:**
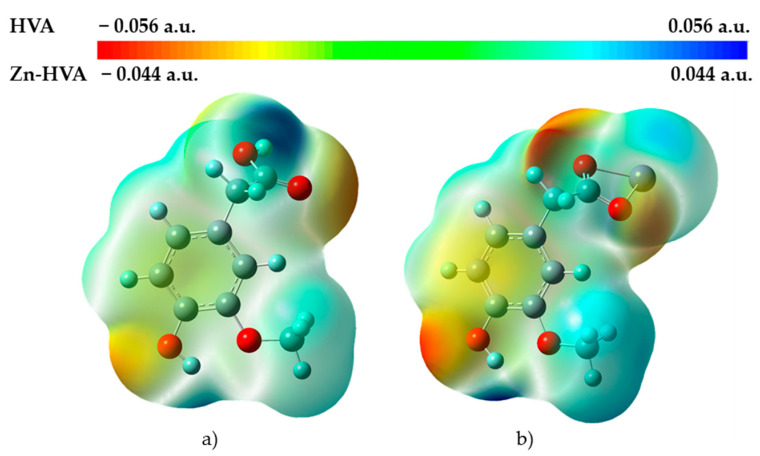
Molecular electrostatic potential maps (MEP) for (**a**) HVA [5]; (**b**) Zn-HVA (in the gas phase).

Molecular electrostatic potential maps (Figure 4) (MEPs) show the electron density and provide a visual method to understand the charged regions as well as the relative polarity of a molecule. Based on these maps, it is possible to represent sites of chemical reactivity of the studied molecules. The MEP on the surface has different values and is shown by different colors [5]. The red region shows the most negative charged positions (electron-rich sites) and corresponds to the regions related to electrophilic reactivity. The blue region indicates the most positive charged positions (electron-deficient areas) and is related to nucleophilic reactivity. The orange, yellow, and green regions indicate slightly negative and positively charged positions, respectively. As one can see from Figure 4, in the case of HVA, the negative potential regions in the map have been spread over oxygen atoms O1 and O2 of the COO^−^ group and phenolic OH group.

It was noticed that after the formation of the Zn-HVA complex, the regions with negative charges did not change, but the amount of negative charge increased (a more intense red color). The positive potential regions (blue color) in the HVA molecule were located over the hydrogen atoms: H9 (of the COOH group) and H4 (of the phenolic OH group).

In the Zn-HVA molecule, the positive values of MEP were localized over the hydrogen atoms: H4 and H3c (of the methoxy group). As can be seen in Figure 4, the Zn atom is a weakly nucleophilic region. The obtained values of the natural charge distribution (NBO), which are presented in Table S3 and Table 2, were consistent with the MEPS data. In fact, as expected, the negative charge was distributed mainly on the oxygen atoms, but in Zn-HVA, the negative charge on the O1 and O2 atoms increased. However, no major changes in the value of the charges on the other atoms or MEP were observed after the formation of Zn-HVA.

#### 3.1.6. Fukui Function (FF) Analysis

The Fukui function can be used to determine the reactivity of each atom in the molecule by analyzing the electron density. It indicates the areas in the molecule susceptible to nucleophilic attack (high f^+^(r) values), electrophilic attack (high f^−^(r) values), and radical attack (high f°(r) values) [28]. If the dual descriptor is $f_{k}^{2}$(r) < 0, then the site is preferred for electrophilic attack, and if it is $f_{k}^{2}$(r) > 0, then it is preferred for nucleophilic attack [29,34]. This analysis was performed for each atom in homovanillic acid and its Zn(II) complex molecules, and the results are shown in Tables S4 and S5. NBO population analysis was used to obtain the Fukui indices.

The obtained Fukui indices suggest that the most preferred sites of nucleophilic attack in the HVA molecule were C6 and H2, while in Zn-HAV, they were C7 and C2. On the other hand, the sites most susceptible to electrophilic attack sites in both the acid molecule and its complex with Zn were C2, C7, and C9. In addition, another place for electrophilic attack was the C8 atom. However, the values of the f^−^(r) and f^+^(r) parameters for Zn-HVA were lower compared to the acid. The most susceptible sites to radical attack in the acid were as follows: H3a, H3c (of the methoxy group), H9 (of the carboxyl group), and C1 and O4 (of the hydroxyl group), while in Zn-HVA, they were Zn, O2, O4, O1, and C4.

### 3.2. UV-Vis Spectroscopy

**Figure 5:**
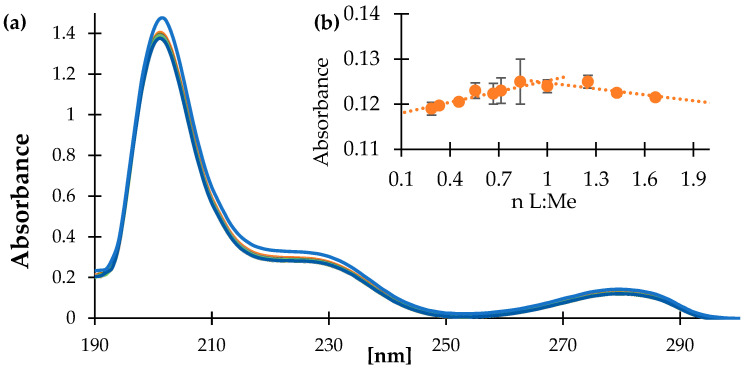
(**a**) The UV/Vis spectra of HVA (blue line; 0.5 mM) and Zn-HVA complexes (max. concentration was 0.20 mM) registered in tris-HCl buffer in the spectral range of 200–550 nm; (**b**) The mole-ratio plot for the composition of Zn-HVA complex (at λ_max_ = 278 nm).

The metal-ligand ratio in the aqueous solution was established based on the spectrophotometric method (Figure 5). In the spectrum of HVA, the three bands were observed at 205, 228, and 278 nm, which slightly decreased in their absorbance after addition of Zn^2+^ ions to the solution. The bands were assigned to π → π* transitions. Based on the change in the absorbance of the band at 278 nm in the series of Zn-HVA aqueous solution (pH = 7.4), the established molar ratio ligand:metal ion was 1:1, and the possible metal ion coordination was via the carboxylate ion. The substituents from the aromatic ring probably do not participate in metal ion coordination in aqueous because there was no distinct movement of the bands assigned to the electronic transitions within the aromatic ring.

### 3.3. FT-IR Spectroscopy

**Figure 6:**
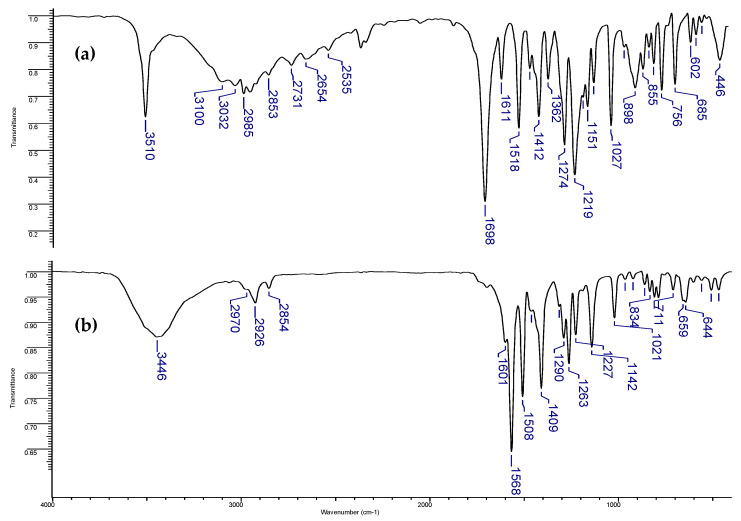
The FT-IR spectra of (**a**) HVA and (**b**) Zn-HVA registered in the spectral range 400–4000 cm^−1^.

In the FT-IR spectrum of HVA, one can observe the characteristic bands assigned to the vibrations of the carboxylic group, as well as the hydroxy and methoxy substituents in the ring (Table S6, Figure 6), i.e., stretching vibrations of the carbonyl group ν(C=O) at 1698 cm^−1^ and the in-plane β(C=O) and out-of-plane γ(C=O) deforming vibrations of the carbonyl group at 756 and 685 cm^−1^ [35]. The above-mentioned bands were not present in the spectrum of Zn-HVA, whereas new ones occurred compared with the spectrum of HVA. Namely, very intense bands associated with asymmetric and symmetric stretching vibrations of the carboxylate anion are found at: ν_as_(COO^−^) at 1568 cm^−1^ and ν_s_(COO^−^) at 1409 cm^−1^, respectively and symmetric in-plane β_as_(COO^−^) and out-of-plane γ(COO^−^) deforming vibrations of the carboxylate anion at 644 cm^−1^ and 789 cm^−1^, respectively. These changes indicate the coordination of the metal ion through the carboxylate anion. Moreover, the slight movement of the corresponding bands from the spectra of HVA and the Zn complex assigned to the vibration of hydroxy and methoxy substituents in the ring suggest the additional coordination of metal ions through the substituents.

### 3.4. NMR

The values of the chemical shifts from the experimental ^1^H and ^13^C NMR spectra of HVA and Zn-HVA are summarized in Table S7. The atom numbering is shown in Figure 2. In the ^1^H NMR spectrum (Figure S2) for the Zn-HVA, the values of the chemical shifts slightly decrease in relation to the appropriate signals in the spectrum of HVA, and there is no signal from the H9 atom, which suggests metal ion coordination through the carboxylate anion. The values of the chemical shifts from the ^13^C NMR (Figure S3) of Zn-HVA assigned to the carbon atoms no. C2, C3, C4, C5, C6, C7, and C9 are lower than the appropriate signals in the spectrum of HVA, whereas the signal from the C1 moved the higher ppm values in the spectrum of Zn-HVA compared to the spectrum of HVA. The coordination of zinc cation through the carboxylate anion caused the disappearance of the signal from the C1 in the spectrum of the complex.

### 3.5. Elemental and Thermogravimetric Analysis

#### 3.5.1. Elemental Analysis

In Table 4, the results of the elemental analysis for the C and H content [wt.%] in the Zn-HVA complex are shown. On the basis of these results, it is concluded that the Zn complex with homovanillic acid possesses the formula Zn_2_(C_9_H_8_O_4_)_3_·2H_2_O, where both carboxylate anion and deprotonated hydroxyl substituent from the aromatic ring participate in metal ion coordination. The metal:ligand molar ratio for the solid complex differed from the one established in aqueous solution. The reason may be more basic pH during the synthesis of the solid complex (the NaOH solution was added to enable metal ion coordination) compared to the neutral pH in the spectrophotometric mole-ratio method.

#### 3.5.2. Thermogravimetric Analysis

**Figure 7:**
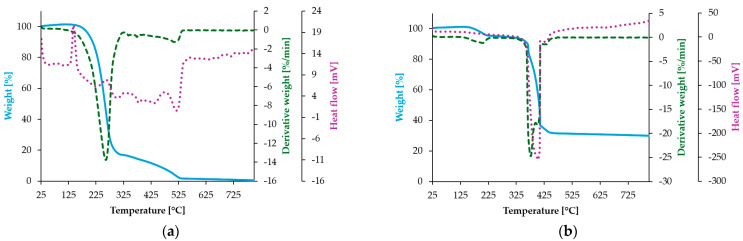
TG (blue), DTG (green), and DSC (violet) curves of (**a**) HVA and (**b**) Zn-HVA.

Homovanillic acid and its Zn(II) complex with the formula Zn_2_(C_9_H_8_O_4_)_3_ ·2 H_2_O were investigated by the TG-DSC thermal method to evaluate the effect of the temperature on the structure of these compounds and to characterize the thermal effects (endothermic or exothermic) associated with the dehydration and oxidation processes. The thermogravimetric curves of HVA and the Zn-HVA complex are presented in Figure 7a,b. The thermal parameters are summarized in Table 5.

The thermal decomposition process of HVA is a two-step process. The first stage is related with the strong endothermic peak at about 160 °C, with a corresponding mass loss of ca. 80 wt. %. The second degradation step is in the temperature range of 300–530 °C. Further thermal degradation occurs with no obtained residue.

The thermal decomposition of the Zn(II) complex occurs in two main stages. The first stage (occurring up to 232 °C) is ascribed to the dehydration of the complex with the formation of an anhydrous compound. It corresponds to the maximum mass loss observed at about 220 °C. In the next stage, rapid decomposition with an oxidation of ligand occurs in a temperature range from 340 °C to 460 °C. The residue after that step consists of ZnO and inorganic by-products. The mass loss at 800 °C is 33.81 wt. %.

### 3.6. Antioxidant Assays

The antioxidant potential of the HVA and Zn-HVA was studied by the Ferric Ion Reducing Antioxidant Power (FRAP), ABTS (diammonium 2,2′-azino-bis(3-ethylbenzothiazoline-6-sulfonate), and DPPH ((1,1-diphenyl-2-picrylhydrazyl) methods. These methods differ in the mechanism of interactions with radicals. In the FRAP assay, one electron is transferred from the antioxidant molecule, which causes the reduction of Fe(III) ions originating from the colored complex with (2,4,6-tris(2-pyridyl)-1,3,5-triazine)—this is the SET mechanism. In turn, the ABTS and DPPH tests are based on a mixed reaction mechanism with free radicals, where both HAT (hydrogen atom transfer) and SET mechanisms are used.

#### 3.6.1. Reducing Activity Assays

The reducing properties determined in the FRAP test are associated with the transfer of an electron from the antioxidant compound to reduce Fe(III) cations. The reducing ac-tivity of the Zn-HVA complex in the FRAP test was slightly higher than that of the acid (but no statistically significant differences were observed), which indicates that complexa-tion of HVA with zinc facilitates electron transfer to a greater extent, which increases its potential as an antioxidant in comparison with the acid.

**Figure 8:**
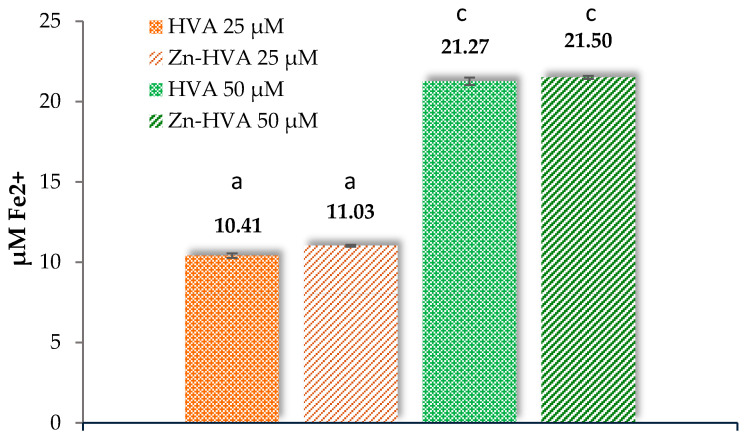
The FRAP values obtained for homovanillic acid (HVA) and Zn(II) complex with homovanillic acid (Zn-HVA). Mean values from three independent experiments ± SD are shown. Different small letters mean statistically significant differences (*p* < 0.05) between the same parameter obtained for HVA and Zn-HVA.

The antioxidant power reducing ion for both the acid and its complex with zinc increased about two times with the increase in the concentrations of these substances (Figure 8). HVA and Zn-HVA showed higher antioxidant activity measured in the FRAP test compared to caffeic acid (a very popular antioxidant and reducing agent). At the same tested concentration, HVA and Zn-HVA showed a higher reducing capacity (Figure 8) than caffeic acid (~15 µM Fe^2+^) [26].

#### 3.6.2. DPPH^•^ Antiradical Activity Assay

**Figure 9:**
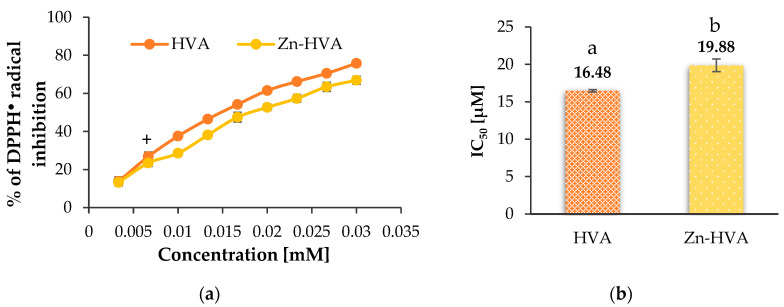
A comparison of the antioxidant activities of HVA and Zn-HVA measured by DPPH assay: (**a**) percentage of inhibition of DPPH^•^ radicals by HVA and its Zn(II) complex (Zn-HVA), depending on their concentration (0.03–0.0033 mM); A plus sign means statistically insignificant differences (*p* > 0.05) between the same parameter obtained for HVA and Zn-HVA. (**b**) The IC_50_ parameter value for HVA and Zn-HVA. Mean values from three independent experiments ± SD are shown. Different small letters mean statistically significant differences (*p* < 0.05) between the same parameter obtained for HVA and Zn-HVA.

The antiradical activity of DPPH^•^ of the studied compounds was expressed as a percentage of DPPH^•^ radical inhibition and as IC_50_ values (Figure 9), which means the concentration of compound that inhibits 50% of the radicals.

The Zn(II) complex of HVA showed slightly lower antiradical activity against DPPH^•^ radicals than the ligand alone. The antiradical properties increased with the increase in the compound concentration. In the studied concentration range (0.0033–0.03 mM), the Zn(II) complex inhibited 13.26–66.93% of the initial DPPH^•^ radicals concentration, whereas HVA inhibited 13.80–75.80%. The IC_50_ values were 19.88 ± 0.85 µM and 16.48 ± 0.16 µM for the Zn-HVA and HVA, respectively. The determined IC_50_ values were higher compared to the IC_50_ values of caffeic acid (by 49.82% and 80.73% for HVA and Zn-HVA, respectively) [26]. A slightly lower IC_50_ value for homovanillic acid (compared to the result received in this study) was obtained by Tuck and Hayball [4] in examining the activity of HVA and its derivatives against the DPPH^•^ radical, when it was 14.8 µM.

#### 3.6.3. ABTS

**Figure 10:**
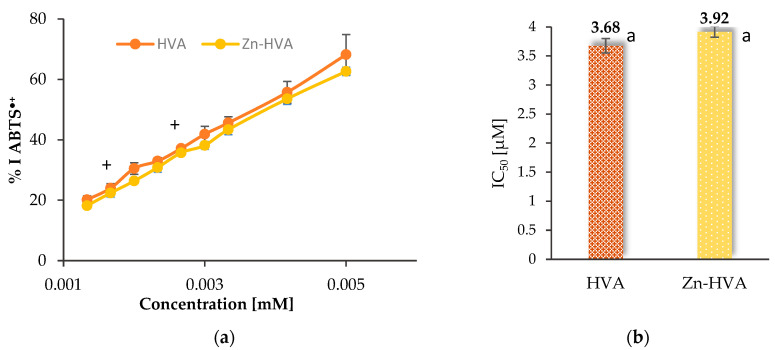
A comparison of the antioxidant activities of the HVA and Zn-HVA measured by ABTS assay: (**a**) percentage of inhibition of ABTS^•+^ radicals by homovanillic acid (HVA) and Zn-HVA, depending on their concentration; a plus sign means statistically insignificant differences (*p* > 0.05) between the same parameter obtained for the acid and Zn(II) complex. (**b**) The IC_50_ parameter value for HVA and Zn-HVA. Mean values from three independent experiments ± SD are shown. The same small letters mean statistically insignificant differences (*p* > 0.05) between the same parameter.

The results of the reactions of the studied compounds with ABTS^•+^ radicals are given as the percentage of ABTS^•+^ radical inhibition and as the IC_50_ parameter (Figure 10). The ABTS test showed that HVA possessed similar antioxidant properties (IC_50_ = 3.68 ± 0.23 μM) to the Zn(II) complex (IC_50_ = 3.92 ± 0.09 μM). In the case of the ABTS^•+^ assay, the statistical test showed there were no statistically significant differences between these results (*p* > 0.05).

The obtained % inhibition of the ABTS^•+^ radical at the maximum analyzed concentration (0.005 mM) was similar for both compounds and amounted to 68.24% and 62.68% for HVA and Zn-HVA, respectively.

### 3.7. Microbiological Studies

**Figure 11:**
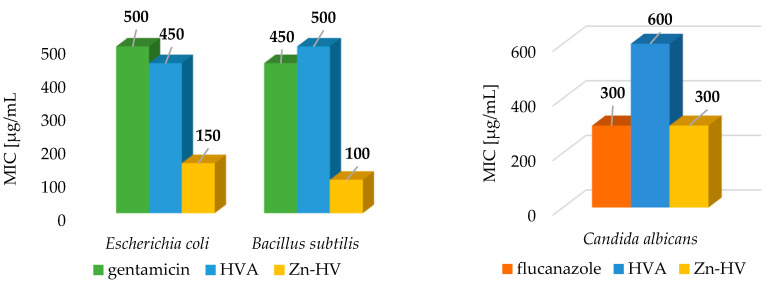
Antimicrobial activity (expressed in MIC values) determined for homovanillic acid (HVA) and the complex of Zn(II) compared to the positive control.

The antibacterial and antifungal activity of homovanillic acid and its complex with Zn(II) against *Escherichia coli*, *Bacillus subtilis*, and *Candida albicans* were investigated by evaluation of the minimum inhibitory concentration (MIC). The results indicated higher antibacterial and antifungal activity of the Zn(II) complex compared to the acid itself (Figure 11). The MIC value obtained in the studies with *C. albicans* for the complex with Zn(II) was two times lower than the value obtained for the acid (MIC = 600 μg/mL), but equal to the value corresponding to the positive control, i.e., flucanazole (MIC = 300 μg/mL). Among all evaluated microorganisms, *C. albicans* showed the highest resistance to all tested compounds. This may be due to (a) the high biofilm-forming capacity of *C. albicans*, which may influence the changes in the availability of the antifungal compound, (b) the high frequency of spontaneous mutations, and (c) overexpression of multidrug efflux pumps [36].

The Zn(II) complex had a much stronger bactericidal effect against both Gram-positive bacteria (*Bacillus subtilis*) and Gram-negative bacteria (*E. coli*). The minimum inhibitory concentrations (MICs) of Zn-HVA were 150 μg/mL and 100 μg/mL for *E. coli* and *Bacillus subtilis*, respectively. These values were significantly lower compared to the positive control (gentamycin). The homovanillic acid was also bactericidal, but the MIC values obtained were comparable to those obtained for gentamycin.

Due to their multifaceted action, more metal complexes with phenolic compounds are effective against more resistant bacteria. One of the reasons for their effective action is the ease of passage of metal ions through bacterial cell walls and the destruction of bacterial cell walls by these ions [37]. The literature data show that zinc ions in bacterial cells are involved in the regulation of cell proliferation and cell differentiation and also participate in enzymatic reactions as cofactors [37,38]. They are also essential in many metabolic processes related to the synthesis and degradation of sugars and proteins. While at lower concentrations Zn^2+^ ions bring beneficial effects, at high concentrations, they inhibit bacterial growth. Studies by Nairn et al. [39] showed that in higher concentrations, zinc cations can compete with other metal ions, which can result in a mismatch of metals in different proteins. As a consequence, this can lead to an imbalance in the bacterial cell.

## 4. Conclusions

A Zn(II) complex of homovanillic acid was synthesized, and the structure was described by NMR, FT-IR, elemental, and thermogravimetric analyses. The vibrational spectra showed that in the Zn-HVA complex, the metal ion was coordinated by carboxylate anion. Moreover, the hydroxy and methoxy substituents in the ring coordinated the metal ion. The participation of the hydroxy group in Zn(II) coordination affected the antioxidant properties of the complex compared to ligand alone. Therefore, in the FRAP, ABTS^•+^, and DPPH^•^ tests (which are based on different antioxidant mechanisms), the HVA showed higher or similar antioxidant activity compared to the complex. The theoretically calculated electronic and thermodynamic parameters supported the discussion on the antioxidant properties. Complexation with zinc ions did not significantly affect the antioxidant properties of the ligand, but the antimicrobial activity of Zn-HVA against *Escherichia coli*, *Bacillus subtilis*, and *Candida albicans* was higher than that of HVA.

## Figures and Tables

**Table 1:** The selected bond lengths [Å] and angles (°) between bonds of homovanillic acid and Zn-HVA complex molecules calculated using B3LYP/6-311++G(d,p).

Atom Numbers ^1^	Distance Between Atoms [Å]	
	HVA [5]	Zn-HVA
C1-C7	1.521	1.519
C7-C8	1.515	1.520
C8-O1	1.207	1.272
C8-O2	1.354	1.269
O1-H9/Zn	2.291	2.128
O2-H9/Zn	0.970	2.12
	**Bond Angles (°)**	
	**HVA [5]**	**Zn-HVA**
C6-C1-C7	120.92	120.74
C7-C1-C2	119.91	120.18
C1-C7-C8	111.28	111.92
C1-C7-H8	109.87	110.07
C1-C7-H7	110.42	110.25
H8-C7-C8	109.53	108.31
H7-C7-C8	106.95	107.41
C7-C8-O1	125.26	119.54
C7-C8-O2	112.25	120.03
C8-O1-H9/Zn	55.22	88.32
C8-O2-H9/Zn	107.20	88.73
C1-C2-2H	119.30	110.17

^1^ The numbering of atoms in the HVA molecule is shown in Figure 2.

**Table 2:** Natural bond orbital (NBO) atomic charges on atoms in molecules of HVA and Zn-HVA.

Atoms ^1^	Charge [e]	
	HVA	Zn-HVA
O1	−0.608	−0.767
O2	−0.685	−0.750
C2	−0.272	−0.273
C3	−0.270	−0.268
C7	−0.483	−0.468
C8	0.815	0.804
H3a	0.177	0.173
H3b	0.173	0.175
H7	0.231	0.228
H9/Zn	0.482	1.333

^1^ The numbering of atoms in the HVA molecule is shown in Figure 2.

**Table 3:** Chemical reactivity and thermodynamical parameters of HVA and Zn-HVA in vacuum solution obtained at the B3LYP/6-311++G(d,p) level of theory.

Parameter	HVA	Zn-HVA
**Chemical reactivity parameters**		
Energy (Hartree *)	−650.08	−2428.81
Dipole moment (Debye)	1.31	2.84
Ionization potential, I = −E_HOMO_ (eV)	6.018	5.999
Electron affinity, A = −A = −E_LUMO_ (eV)	0.719	1.139
Electronegativity, $\chi =\frac{I+A}{2}$ (eV)	3.369	3.569
Electronic chemical potential, $\mu =-\frac{I+A}{2}$ (eV)	−3.369	−3.569
Chemical hardness, $\eta =\frac{I-A}{2}$ (eV)	2.650	2.430
Chemical softness, S $=\frac{1}{2\eta }$ (eV)	0.189	0.206
Electrophilicity index, $\omega =\frac{\mu^{2}}{2\eta }$ (eV)	2.141	2.620
**Thermodynamical parameters (kcal/mol)**		
Bond dissociation enthalpy, BDE	95.16	104.31
Ionization potential, IP	185.46	171.54
Proton dissociation enthalpy, PDE	223.80	246.88
Proton affinity, PA	359.67	344.75
Electron transfer enthalpy, ETE	49.59	73.67

* 1 Hartree = 2625.5 kJ/mol.

**Table 4:** Results of the elemental analysis of HVA and Zn-HVA complex.

Compound	C [wt. %]		H [wt. %]	
	Calc	Exp	Calc	Exp
Zn_2_(C_9_H_8_O_4_)_3_·2H_2_O	45.85	45.77 ± 2.01	3.99	3.57 ± 0.19

**Table 5:** Results of the thermal analysis of HVA and the Zn-HVA complex.

Compound	Stage	TG	DTG (DSC)	Process Nature	Mass Loss [wt. %]		Residue
		T_range_ [°C]	T_max_ (Peaks) [°C]		Calc	Exp	
HVA	Decomposition	165–535	261 (265)	Egzo	100	99.5	-
Zn-HVA	Dehydration	150–232	206	Endo	5.09	4.88	Zn_2_C_27_H_24_O_12_
	Decomposition	340–460	376 (389) 402 (403) 434 (434)	egzo egzo egzo	71.89	61.72	ZnO; inorganic residue

## Data Availability

The original contributions presented in the study are included in the article, further inquiries can be directed to the corresponding author.

## Supplementary Materials

The following supporting information can be downloaded at: https://www.mdpi.com/article/10.3390/ma18102374/s1. Figure S1: The numbering of atoms in the HVA molecule; Figure S2: ^1^H NMR spectra of HVA (a) and its Zn(II) complex (b); Figure S3: ^13^C NMR spectra of HVA (a) and its Zn(II) complex(b); Table S1: The bond lengths of homovanillic acid and complex of Zn(II) molecules calculated using DFT/B3LYP/6-311++G(d,p); Table S2: The bond angles of homovanillic acid and complex of Zn(II) molecules calculated using DFT/B3LYP/6-311++G(d,p); Table S3: Data of NBO atomic charge analysis for HVA and complex of Zn(II); Table S4: The values of NBO natural charge, condensed Fukui functions: f^+^, f^−^, (f°), and the dual descriptor for homovanillic acid at the DFT/ B3LYP/6-311++G(d,p) level of theory in the gas phase. Table S5: The values of NBO natural charge, condensed Fukui functions: f^+^, f^−^, (f°), and the dual descriptor for the Zn-HVA complex at the DFT/ B3LYP/6-311++G(d,p) level of theory in the gas phase; Table S6: The wave numbers [cm^−1^], intensities (Int.), and assignments of the bands from the FT-IR spectra of HVA and Zn-HVA; Table S7:The chemical shifts (ppm) from the experimental ^1^H and ^13^C NMR spectra of HVA and Zn-HVA.
