# Supplementary material for: Zn Complex with Homovanillic Acid: Theoretical (B3LYP/6-311++G(d,p)), Structural (FT-IR, NMR), Thermal (TG, DTG, and DSC) and Biological (Antioxidant and Antimicrobial) Characteristics
Source: Materials (Basel). 2025 May 20;18(10):2374. doi: 10.3390/ma18102374 (PMC12113428; doi:10.3390/ma18102374)
Supplement: Supplementary file 1 [file materials-18-02374-s001.zip › materials-3584863-supplementary.pdf]

Supplementary Materials

Zn Complex with Homovanillic Acid: Theoretical (B3LYP/6-311++G(d,p)), Structural (FT-IR, NMR), Thermal (TG, DTG, and DSC) and Biological (Antioxidant and Antimicrobial) Characteristics

Mariola Samsonowicz [1,*], Monika Kalinowska [1], Adriana Dowbysz [1], Kamila Koronkiewicz [1], Bożena Kukfisz [2] and Anna Pietryczuk [3]

1 Department of Chemistry Biology and Biotechnology, Bialystok University of Technology, Wiejska 45E, 15-351 Bialystok, Poland
2 Institute of Safety Engineering, Fire University, Slowackiego Street 52/54, 01-629 Warsaw, Poland
3 Department of Water Ecology, Faculty of Biology, University of Bialystok, Ciołkowskiego 1J, 15-245 Bialystok, Poland
* Correspondence: m.samsonowicz@pb.edu.pl

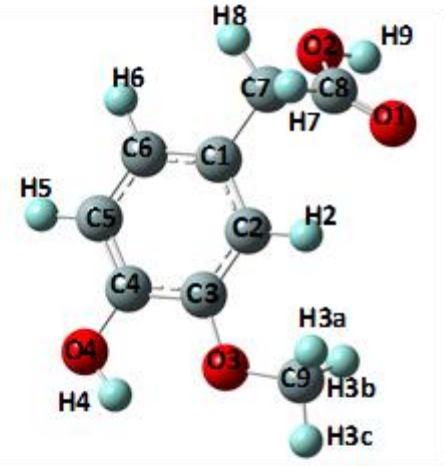

Figure S1. The numbering of atoms in the HVA molecule

Table S1. The bond lengths of homovanillic acid and complex of Zn(II) molecules calculated using DFT/B3LYP/6-311++G(d,p).

Atom numbers	Distance Between Atoms [Å]	
	HVA	$Zn(HVA)^{+}$
C1-C2	1.404	1.404
C2-C3	1.388	1.388
C3-C4	1.408	1.408
C4-C5	1.386	1.386
C5-C6	1.397	1.397
C6- C1	1.392	1.393
C1-C7	1.521	1.519
C7-C8	1.515	1.52
C8-O1	1.207	1.272
C8-O2	1.354	1.269
O1-H9/Zn	2.291	2.128
O2-H9/Zn	0.970	2.12
C3-O3	1.372	1.373
O3-C9	1.424	1.424
C4-O4	1.362	1.363
O4-H4	0.967	0.967
C7-H7	1.093	1.093
C7-H8	1.090	1.089
C2-H2	1.082	1.082
C9-H3a	1.094	1.095
C9-H3b	1.095	1.094
C9-H3c	1.089	1.089
C5-H5	1.083	1.083
C6-H6	1.084	1.084

Table S2. The bond angles of homovanillic acid and complex of Zn(II) molecules calculated using DFT/B3LYP/6-311++G(d,p).

Atom numbers	*Bond angles* (°)	
	HVA	$ZnHVA^+$
C1-C2-C3	120.13	120.16
C2-C3-C4	120.35	120.37
C3-C4-C5	119.48	119.46
C4-C5-C6	120.08	120.08
C5-C6- C1	120.79	120.84
C6- C1- C2	119.17	119.09
C6- C1-C7	120.92	120.74
C7-C1-C2	119.91	120.18
C1-C7-C8	111.28	111.92
C1-C7-H8	109.87	110.07
C1-C7-H7	110.42	110.25
H8-C7-H7	108.72	108.77
H8-C7-C8	109.53	108.31
H7-C7-C8	106.95	107.41
C7-C8-O1	125.26	119.54
C7-8C-O2	112.25	120.03
C8-O1-H9/Zn	55.215	88.32
C8-O2-H9/Zn	107.20	88.73
C2-C3-O3	125.81	125.78
O3-C3-C4	113.84	113.84
C3-O3-C9	118.60	118.52
C3-C4-O4	120.23	120.26
C5-C4-O4	120.29	120.28
C4-O4-H4	107.77	107.76
C1-C2-H2	119.30	119.17
H2-C2-C3	120.57	120.66
O3-C9-H3a	111.08	111.03
O3-C9-H3b	111.02	111.06
O3-C9-H3c	105.96	105.99
C4-C5-H5	118.74	118.75
H5-C5-C6	121.17	121.17
C5-C6-H6	119.32	119.32
H6-C6- C1	119.89	119.83

Table S3. Data of NBO atomic charge analysis for HVA and complex of Zn(II).

Atom numbers	Charge (e^1)	
	HVA	$ZnHVA^+$
O1	-0.608	-0.767
O2	-0.685	-0.75
O3	-0.570	-0.571
O4	-0.673	-0.674
C1	-0.049	-0.048
C2	-0.272	-0.271
C3	0.270	0.268
C4	0.285	0.284
C5	-0.237	-0.238
C6	-0.203	-0.202
C7	-0.483	-0.468
C8	0.815	0.804
C9	-0.202	-0.201
H2	0.223	0.224
H3a	0.177	0.173
H3b	0.173	0.175
H3c	0.191	0.191
H4	0.485	0.485
H5	0.218	0.218
H6	0.205	0.206
H7	0.231	0.228
H8	0.228	0.228
H9/Zn	0.482	0.707

Table S4. The values of NBO natural charge, condensed Fukui functions: f+, f-, (f0) and dual descriptor for homovanillic acid at the DFT/ B3LYP/6-311++G(d,p) level of theory in gas-phase.

	NBO natural charge			Fukui function			
Atoms numbering	$q_{(N+1)}$	$q_{(N0)}$	$q_{(N-1)}$	$f_j^+(r)$	$f_j^-(r)$	f_j^0	$f^2(r)$
O1	-0.618	-0.608	-0.556	-0.01	-0.052	0.031	0.042
O2	-0.715	-0.685	-0.666	-0.03	-0.019	0.0245	-0.011
O3	-0.578	-0.57	-0.478	-0.008	-0.092	**0.05**	0.084
O4	-0.689	-0.673	-0.540	-0.016	-0.133	**0.0745**	0.117
C1	-0.049	-0.049	0.108	0	-0.157	**0.0785**	0.157
C2	-0.273	-0.272	-0.279	-0.001	**0.007**	-0.003	-0.008
C3	0.271	0.27	0.359	0.001	-0.089	0.044	0.09
C4	0.277	0.285	0.391	-0.008	-0.106	**0.057**	0.098
C5	-0.249	-0.237	-0.211	-0.012	-0.026	0.019	0.014
C6	0.212	-0.203	-0.112	**0.415**	-0.091	-0.162	0.506
C7	-0.503	-0.483	-0.508	-0.02	**0.025**	-0.0025	-0.045
C8	0.799	0.815	0.805	-0.016	**0.01**	0.003	-0.026
C9	-0.233	-0.202	-0.219	-0.031	**0.017**	0.007	-0.048
H2	0.294	0.223	0.257	**0.071**	-0.034	-0.0185	0.105
H3a	0.064	0.177	0.199	-0.113	-0.022	**0.0675**	**-0.091**
H3b	0.121	0.173	0.201	-0.052	-0.028	0.04	-0.024
H3c	-0.028	0.191	0.223	-0.219	-0.032	**0.1255**	**-0.187**
H4	0.462	0.485	0.507	-0.023	-0.022	0.0225	-0.001
H5	0.198	0.218	0.258	-0.02	-0.04	0.03	0.02
H6	0.174	0.205	0.241	-0.031	-0.036	0.0335	0.005
H7	0.140	0.231	0.268	-0.091	-0.037	**0.064**	-0.054
H8	0.156	0.228	0.250	-0.072	-0.022	0.047	-0.05
H9	0.281	0.482	0.501	-0.201	-0.019	**0.11**	**-0.182**

Table S5. The values of NBO natural charge, condensed Fukui functions: f+, f-, (f^0) and dual descriptor for Zn-HVA complex at the DFT/ B3LYP/6-311++G(d,p) level of theory in gas-phase.

	NBO natural charge			Fukui function			
Atoms numbering	$q_{(N+1)}$	$q_{(N0)}$	$q_{(N-1)}$	$f_j^+(r)$	$f_j^-(r)$	f_j^0	$f^2(r)$
O1	-0.798	-0.767	-0.712	-0.086	-0.055	0.043	0.024
O2	-0.779	-0.75	-0.688	-0.091	-0.062	0.0455	0.033
O3	-0.581	-0.571	-0.53	-0.051	-0.041	0.0255	0.031
O4	-0.691	-0.674	-0.601	-0.09	-0.073	0.045	0.056
C1	-0.022	-0.048	-0.001	-0.021	-0.047	0.0105	0.073
C2	-0.265	-0.271	-0.275	0.01	0.004	-0.005	0.002
C3	0.26	0.268	0.308	-0.048	-0.04	0.024	0.032
C4	0.265	0.284	0.349	-0.084	-0.065	0.042	0.046
C5	-0.247	-0.238	-0.22	-0.027	-0.018	0.0135	0.009
C6	-0.207	-0.202	-0.154	-0.053	-0.048	0.0265	0.043
C7	-0.476	-0.468	-0.47	-0.006	0.002	0.003	-0.01
C8	0.775	0.804	0.812	-0.037	-0.008	0.0185	-0.021
C9	-0.201	-0.201	-0.209	0.008	0.008	-0.004	-0.008
H2	0.23	0.224	0.235	-0.005	-0.011	0.0025	0.017
H3a	0.169	0.173	0.186	-0.017	-0.013	0.0085	0.009
H3b	0.185	0.175	0.18	0.005	-0.005	-0.0025	0.015
H3c	0.178	0.191	0.212	-0.034	-0.021	0.017	0.008
H4	0.48	0.485	0.499	-0.019	-0.014	0.0095	0.009
H5	0.205	0.218	0.244	-0.039	-0.026	0.0195	0.013
H6	0.201	0.206	0.225	-0.024	-0.019	0.012	0.014
H7	0.213	0.228	0.261	-0.048	-0.033	0.024	0.018
H8	0.212	0.228	0.251	-0.039	-0.023	0.0195	0.007
H9	-0.106	0.707	1.098	-1.204	-0.391	0.602	-0.422

Table S6. The wavenumbers [cm^{-1}], intensities (Int.) and assignments of the bands from the FT-IR spectra of HVA and Zn-HVA.

HVA		Zn-HVA		Assignments
Wavenumber [cm^{-1}]	Int.	Wavenumber [cm^{-1}]	Int.	
3510	m	3446	m	$\nu(OH)_{ar}$
1698	vs	-		$\nu(C{=}O)_{COOH}$
1611	m	1601	m	$\nu(CC)$
-		1568	vs	$\nu_{as}(COO^-)$
1518	s	1508	S	$\nu(CC)$
1458	w	1462	w	$\delta_{as}(CH_3)$
1438	m	-		$\delta_{as}(CH_3)$
1412	m	-		$\nu(CC)$
-		1409	s	$\nu_s(COO^-)$
1362	m	-		$\delta_s(CH_3)$
1274	s	1263	m	$\nu C{-}(OH)_{ar}$
1219	s	1227	m	$\nu O\text{-}(CH_3)$
1151	m	1142	m	$\beta(CH)$
1027	m	1021	w	$\delta_{as}(CH_3)$
898	m	-		$\gamma(OH)$
-		789	w	$\gamma_s(COO)$
756	m	-		$\beta(C{=}O)$
685	m	-		$\gamma(C{=}O)$
-		644	w	$\beta_{as}(COO)$

[1] vs – very strong; s – strong; m – medium; w – week; vw – very week; ν – stretching vibrations; β –in-plane deforming vibrations; γ – out-of-plane deforming vibrations; δ – scissoring vibrations; as – asymmetric vibrations; s – symmetric vibrations.

Table S7. The chemical shifts (ppm) from the experimental ^{1}H and ^{13}C NMR spectra of HVA and Zn-HVA.

Atoms	Chemical shifts		Atoms	Chemical shifts	
	HVA	Zn-HVA		HVA	Zn-HVA
H2	6.810	6.800	C1	125.759	130.00
H5	6.699	6.685	C2	113.548	113.515
H6	6.635	6.626	C3	147.347	147.274
H3a	3.735	3.733	C4	145.294	145.220
H3b	3.736	3.733	C5	115.289	115.207
H3c	3.736	3.733	C6	121.690	121.604
H4	-	-	C7	40.326	39.520
H7	3.421	3.414	C8	173.148	-
H8	3.421	3.414	C9	55.599	55.554
H9	8.798	-			

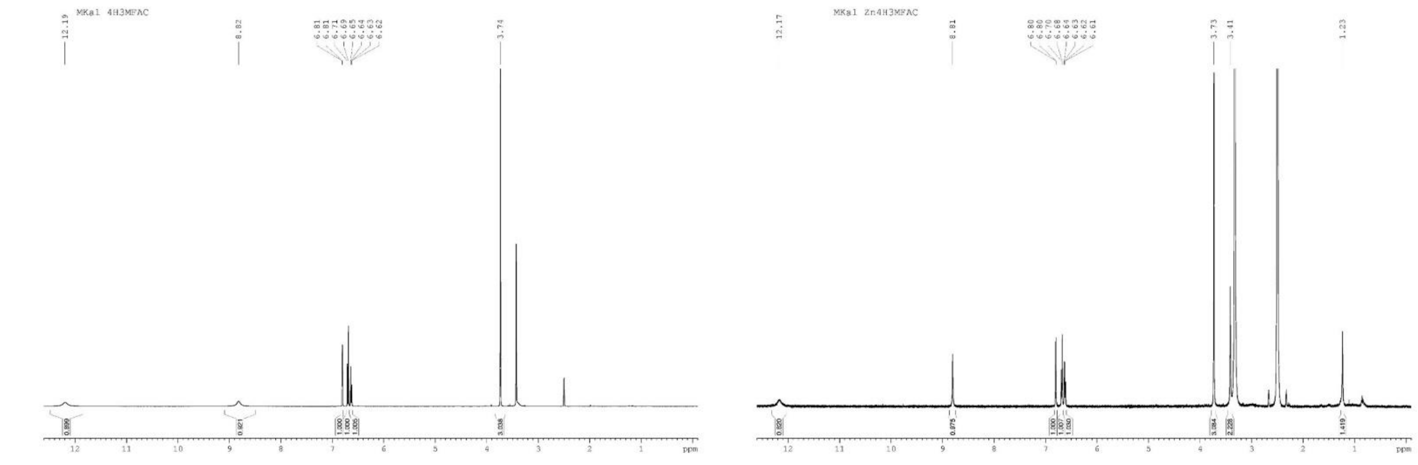

a) b)

Figure S2. ^{1}H NMR spectra of HVA (a) and its Zn(II) complex(b).

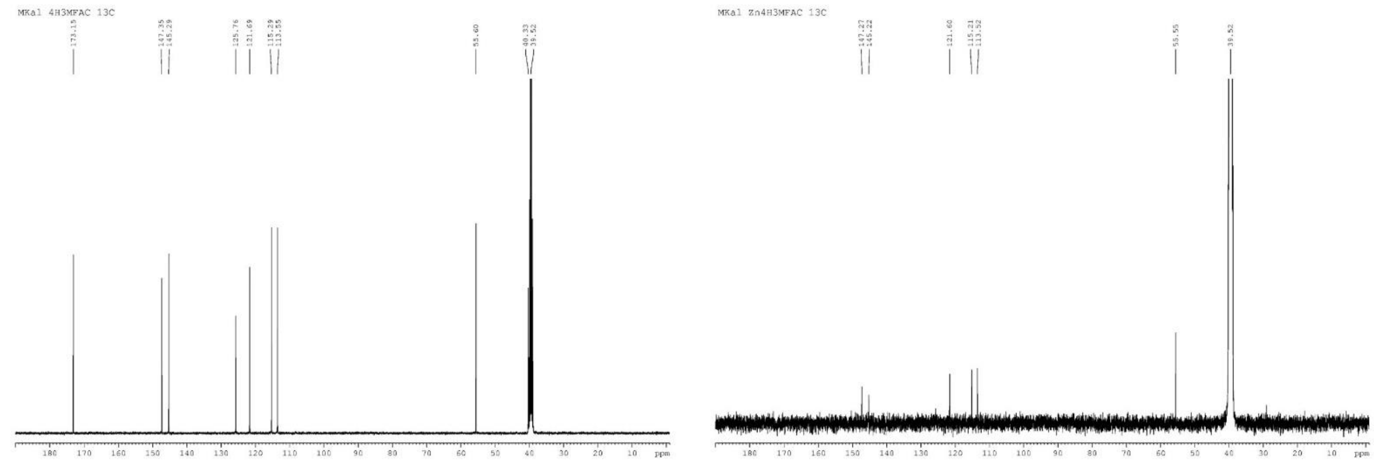

a) b)

Figure S3. ^{13}C NMR spectra of HVA (a) and its Zn(II) complex(b).
